# Comparison of Dimensional Accuracy of Diagnostic Trial Restoration Transfer with Four Different Methods: A Randomized Clinical Trial

**DOI:** 10.3390/jcm14093240

**Published:** 2025-05-07

**Authors:** Lucas Queiroz Caponi, Pilar Fenoy-Illacer, Oscar Figueras-Álvarez, Eduardo de Lima Flor, Carla Vidal-Ponsoda, Miguel Roig

**Affiliations:** 1 Department of Restorative Dentistry, School of Dentistry, Hospital Universitari General de Catalunya, International University of Catalunya, Carrer Josep Trueta s/n, 08195 San Cugat del Vallés, Spain; l.queirozcaponi@uic.es (L.Q.C.); pilarfenoy@uic.es (P.F.-I.); ofigueras@uic.es (O.F.-Á.); eduardorafaelflor@gmail.com (E.d.L.F.); carlavidal11@uic.es (C.V.-P.); 2 Private Practice, 63074 San Benedetto del Tronto, Italy

**Keywords:** CAD-CAM dentistry, computer-aided design, computer-aided manufacturing, dental aesthetics, dental prosthesis design, three-dimensional printing

## Abstract

**Background/Objective:** Diagnostic trial restorations play a crucial role in restorative dentistry by allowing clinicians to evaluate aesthetics, function, and phonetics before finalizing definitive restorations. These restorations facilitate communication between patients, clinicians, and dental technicians, ensuring treatment alignment and predictable outcomes. The accuracy of transferring diagnostic trial restorations to the oral cavity is essential to maintain the integrity of the planned design. Various fabrication techniques, including conventional silicone matrices and computer-aided design/computer-aided manufacturing (CAD-CAM)-based methods, have been developed to improve transfer precision. However, there is limited evidence directly comparing their dimensional accuracy. This randomized in vivo study aimed to evaluate and compare the accuracy of four commonly used techniques—condensation silicone, addition PVS silicone, transparent PVS silicone, and CAD-CAM combination matrices—by assessing their linear and volumetric discrepancies. **Methods**: Twenty patients requiring aesthetic rehabilitation of their anterior maxillary teeth participated. The sequence of matrix usage was determined through randomization. Four techniques for transferring diagnostic trial restorations were evaluated: (1) condensation silicone matrix, (2) addition polyvinyl siloxane (PVS) silicone matrix, (3) transparent PVS silicone matrix, and (4) CAD-CAM combination matrix. Dimensional accuracy was assessed by comparing intraoral scans (IOSs) of the transferred restorations to the original diagnostic wax-up. Linear discrepancies were measured at four buccal landmarks (cervical, medial, lower medial, and incisal), and volumetric deviation was evaluated using reverse engineering alignment software. **Results:** Significant differences were observed among the groups in both linear and volumetric discrepancies (*p* < 0.05). The CAD-CAM combination matrix showed superior volumetric accuracy, with minimal deviations from the diagnostic wax-up. The addition PVS silicone matrix demonstrated consistent linear accuracy, particularly at the cervical and medial landmarks. The condensation silicone matrix exhibited moderate performance across both linear and volumetric accuracy. The transparent PVS silicone matrix showed the highest variability, with greater volumetric deviations. **Conclusions:** The study highlights that the choice of matrix material and technique significantly impacts the dimensional accuracy of diagnostic trial restoration transfers. The CAD-CAM combination matrix and the addition PVS silicone matrix demonstrated superior advantages compared to the other techniques. Clinicians should consider the specific requirements of each case, including accuracy and ease of use, when selecting a transfer technique for aesthetic rehabilitations.

## 1. Introduction

Restorative dentistry has increasingly emphasized the role of diagnostic trial restorations in achieving predictable outcomes in anterior aesthetic rehabilitations [1,2,3,4]. Commonly referred to as aesthetic prototypes, diagnostic trial restorations allow clinicians to evaluate critical parameters such as aesthetics, function, and phonetics, before finalizing definitive restorations [5,6]. They also facilitate effective communication among patients, clinicians, and dental technicians, ensuring alignment in treatment goals and expectations. By accurately replicating the planned restoration design, diagnostic trial restorations play a pivotal role in optimizing treatment outcomes and minimizing the likelihood of complications [7,8,9,10].

The process of transferring diagnostic trial restorations to the oral cavity demands precision and reproducibility, as dimensional accuracy is essential for maintaining the integrity of the original wax-up cast design [5]. Inaccurate transfers may result in deviations in shape, position, or fit, potentially compromising the success of the final restoration. To address these challenges, various techniques and materials have been developed, each offering advantages and limitations.

Traditionally, indirect methods using matrices to mold the diagnostic waxed cast for transferring trial restorations have been adopted. Materials such as vacuum-formed plastic shells, condensation silicone, and addition polyvinyl siloxane (PVS) silicone have been extensively used for this purpose [11]. Nevertheless, concerns regarding material deformation, operator variability, and insufficient rigidity have raised questions about the reliability of these conventional methods.

Advancements in computer-aided design/computer-aided manufacturing (CAD-CAM) technology have introduced more sophisticated approaches to matrix fabrication. Flexible 3D-printed resins have emerged as an alternative for fabricating entire trial restoration matrices, providing a modern solution [12]. However, these resins often fail to replicate the fine details of the diagnostic wax-up as effectively as PVS-based silicones. Direct techniques, which involve using printed or milled prototype restorations to simulate the final design, have also gained popularity [13]. Nevertheless, these methods are particularly suited for cases with adequate restorative material thickness and are primarily effective in additive scenarios [14,15,16].

Recently, hybrid techniques have been introduced that combine CAD-CAM fabricated rigid matrices with PVS-based silicone relining [17,18]. This hybrid approach seeks to enhance transfer accuracy by reducing matrix deformation while preserving the intricate details of the diagnostic wax-up.

Despite these advancements, there is limited evidence directly comparing the dimensional accuracy of these techniques in transferring diagnostic trial restorations. This randomized clinical trial (RCT) aimed to evaluate and compare four techniques for transferring diagnostic trial restorations: condensation silicone, addition PVS silicone, transparent PVS silicone, and CAD-CAM combination matrices. By assessing linear and volumetric discrepancies in vivo, this RCT hypothesizes that differences in material properties and fabrication techniques influence the accuracy of diagnostic trial restoration transfers, potentially impacting clinical outcomes in anterior aesthetic rehabilitations.

## 2. Materials and Methods

This was a single-center, within-subject randomized clinical trial in which each participant received all four intervention techniques. The order of matrix application was randomized using a computer-generated sequence.

**Figure 1:**
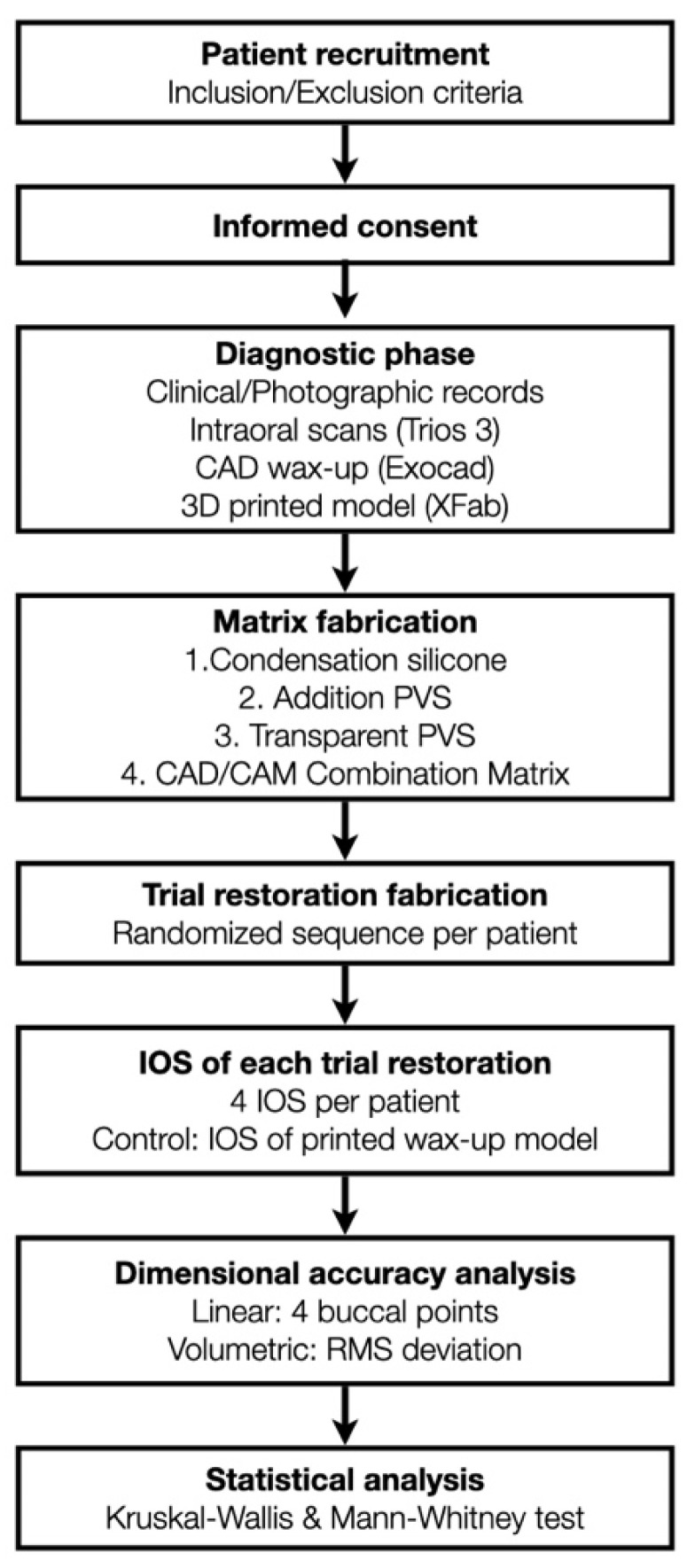
Flow chart illustrating the complete clinical and laboratory workflow of the randomized clinical trial. The diagram summarizes patient recruitment, diagnostic procedures, matrix fabrication protocols, trial restoration transfer, intraoral scanning, and digital analysis steps. IOS = intraoral scanning; STL = standard tessellation language; PVS = polyvinyl siloxane; CAD = computer-aided design; CAM = computer-aided manufacturing; RMS = root mean square.

This RCT was approved by the Ethical Committee for Medicinal Research (CEIM) of the International University of Catalunya. Patients were recruited from the Clínica Universitaria Odontológica (CUO) and selected based on the need for aesthetic rehabilitation of the maxillary anterior teeth. Participants were enrolled between April 2020 and June 2021; all procedures were completed within the same clinical session. All participants provided informed consent after receiving detailed information about the study procedures. An overview of the full clinical workflow and study design is illustrated in Figure 1.

### 2.1. Inclusion Criteria

- Patients aged 18–70 years;
- Intact maxillary anterior teeth (#13–#23);
- Need for additive restorative rehabilitation for buccal and incisal coverage.

### 2.2. Exclusion Criteria

- Presence of removable prostheses;
- Missing maxillary anterior teeth;
- Significant buccal inclination of any anterior tooth.

A total of 20 patients were enrolled in the study. A pilot study with five patients was conducted to estimate mean values and standard deviations for dimensional accuracy. Based on these data, a sample size calculation using the Statulator tool indicated that a minimum of 13 patients was required. To ensure adequate power, 20 patients were included.

Each patient underwent a standardized diagnostic phase that included clinical and radiographic examinations, intraoral and extraoral photography, and intraoral scanning (IOS) with a calibrated intraoral scanner (Trios3, 3Shape, Copenhagen, Denmark). Diagnostic wax-ups were digitally designed using CAD software (DentalCAD 3.0 Galway, exocad GmbH, Darmstadt, Germany) based on the IOS data and fabricated using additive manufacturing (XFAB 2000, DWS, Thiene, Italy) with a model resin (3D-printable resin Invicta 915; DWS, Thiene, Italy). Printed casts underwent post-processing that included an ultrasonic alcohol bath (96%) for 5 min, drying, and curing in an ultraviolet curing chamber for 7 min (Wash & Cure machine 2.0, Anycubic 3D Printing, Shenzhen, China). After processing, the diagnostic wax-up casts were scanned using IOS (Trios3, 3Shape, Copenhagen, Denmark) to obtain reference STL files, which served as the control group for the study.

Simple randomization without restrictions was used. The allocation sequence for matrix order was generated prior to clinical procedures and was concealed from the operator until the day of restoration to reduce potential bias. The random sequence was generated by a researcher not involved in treatment. The same clinician enrolled participants, performed all procedures, and followed the randomized sequence. No changes to methods were made after the trial commenced.

### 2.3. Matrix Fabrication

**Figure 2:**
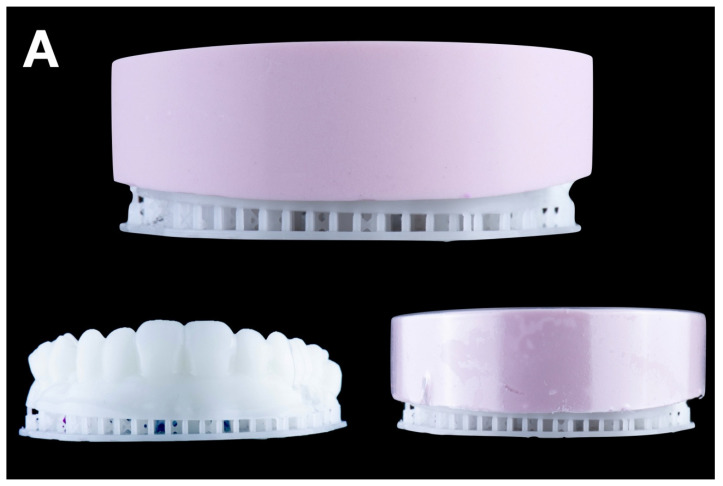

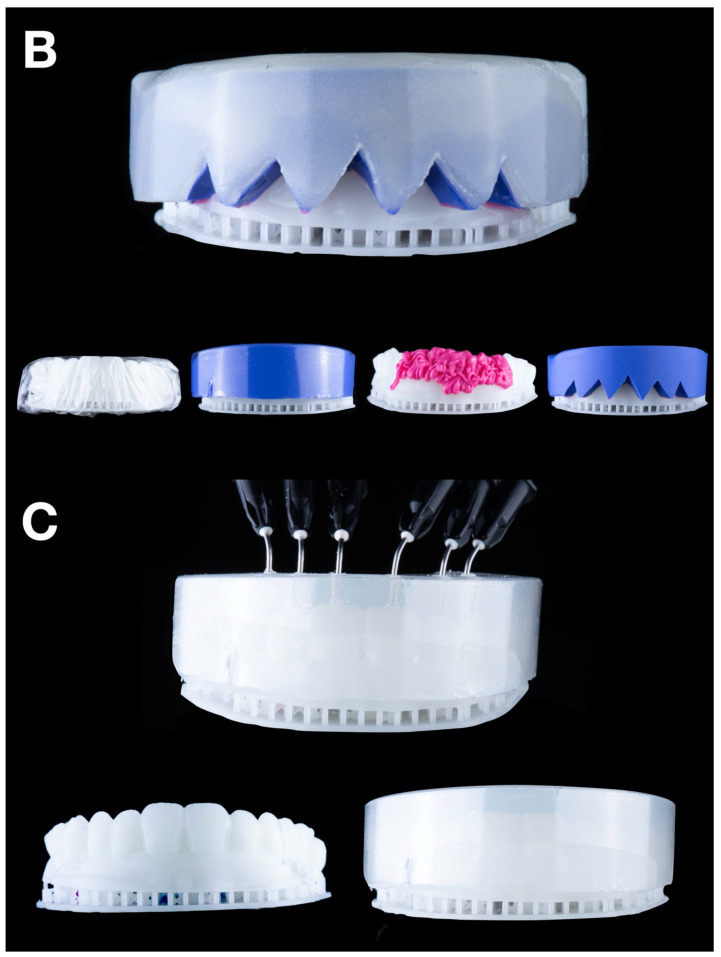

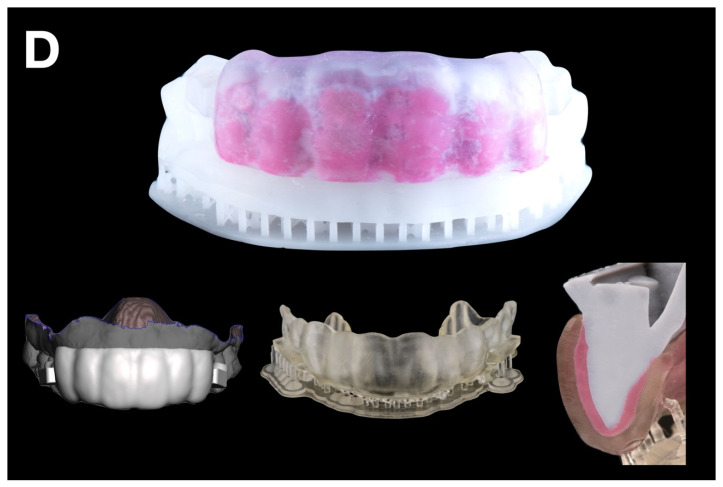
Mock-up matrices fabrication: (**A**) condensation silicone matrix; (**B**) addition PVS silicone matrix; (**C**) transparent addition PVS silicone matrix; (**D**) CAD/CAM combination matrix.

Four types of matrices were fabricated from the diagnostic waxed casts:

**Condensation silicone matrix**: a condensation silicone material (Zetalabor, Zhermack SpA, Badia Polesine, Italy) was manually applied to the wax-up cast, ensuring an overall distribution of 8–12 mm in thickness. After setting, following the manufacturer’s recommendations, the matrix was repositioned onto the wax-up model and evenly trimmed using a dental model trimmer (Dual wheel dental model trimmer, Dentaurum Inc., Ispringen, Germany) until a uniform thickness of 6 mm was achieved. Finally, with the trimmed matrix still positioned on the wax-up model, a vacuum shell was fabricated over it to replicate material distribution and thickness consistently across all matrix types (Figure 2A).

**Addition PVS silicone matrix**: a two-step putty-wash technique using addition PVS silicone (Hydrorise Putty, Zhermack SpA, Badia Polesine, Italy) was employed.

A plastic film spacer (0.25 mm) was placed over the wax-up cast, and putty was packed into the vacuum shell. The loaded vacuum shell was then carefully transferred onto the wax-up cast, ensuring even material distribution and adaptation. After setting, the spacer was removed, and light silicone (Hydrorise Light, Zhermack SpA, Badia Polesine, Italy) was used to reline the matrix. Finally, a resin-based shell (Triad light-curing trays, Dentsply International Inc. York, PA, USA) was applied and cured for added rigidity (Figure 2B).

**Transparent PVS silicone matrix**: transparent addition PVS silicone (Elite Glass, Zhermack SpA, Badia Polesine, Italy) was poured into the vacuum shell and relined over the wax-up cast. After it set, holes were drilled into the incisal areas of each tooth using a bur to insert tips of flowable composite resin during the transfer process (Figure 2C).

**CAD-CAM combination matrix**: a rigid, biocompatible resin matrix was designed (DentalCAD 3.0 Galway, exocad GmbH, Darmstadt, Germany) and 3D printed (Phrozen Mini 4K, Phrozen Tech Co. LTD., Hsinchu, Taiwan) using transparent biocompatible resin (Dental Clear, Harz Labs, Moscow, Russia). After printing, the matrices were subjected to a post-processing protocol recommended by the manufacturer. This included immersion in 96% isopropyl alcohol within an ultrasonic bath for 5 min to remove any residual uncured resin. Subsequently, the matrices underwent a dual-stage light-curing cycle using a UV curing chamber (Wash & Cure 2.0, Anycubic 3D Printing, Shenzhen, China). In the first stage, the printed matrices were cured for 7 min. In the second stage, the matrices were immersed in glycerin gel and exposed to an additional 7 min of UV light to ensure complete polymerization and eliminate the oxygen-inhibited surface layer. This procedure ensured optimal mechanical and dimensional stability of the printed matrices. The matrix provided a calibrated, uniformly distributed space for low-shore PVS silicone material (Hydrorise Light, Zhermack SpA, Badia Polesine, Italy), which was used to reline the matrix. Buccal openings and distal stops ensured proper fit and visual control during relining (Figure 2D).

### 2.4. Trial Restoration Fabrication

Each matrix was used to fabricate diagnostic trial restorations with the following materials: for the condensation silicone matrix, addition PVS silicone matrix, and CAD-CAM combination matrix, auto-polymerization bisacrylic resin (Luxatemp Star, DMG, Hamburg, Germany) was used. For the transparent PVS silicone matrix, light-cured flowable composite resin (Filtek Supreme Flow, 3M Dental Products, Saint Paul, MN, USA) was employed.

**Figure 3:**
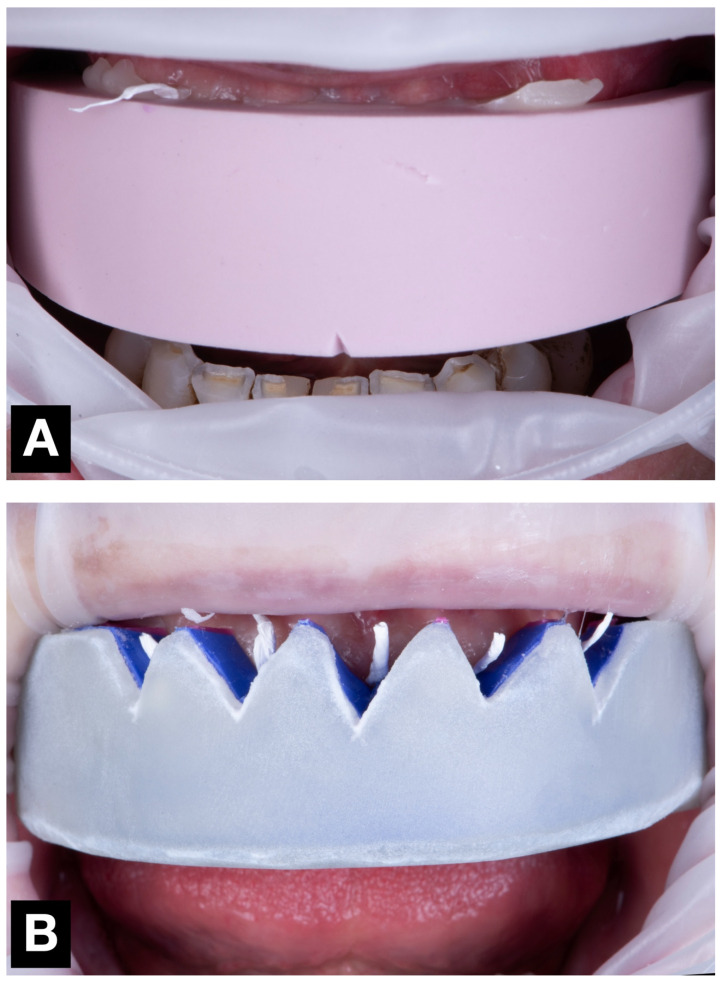

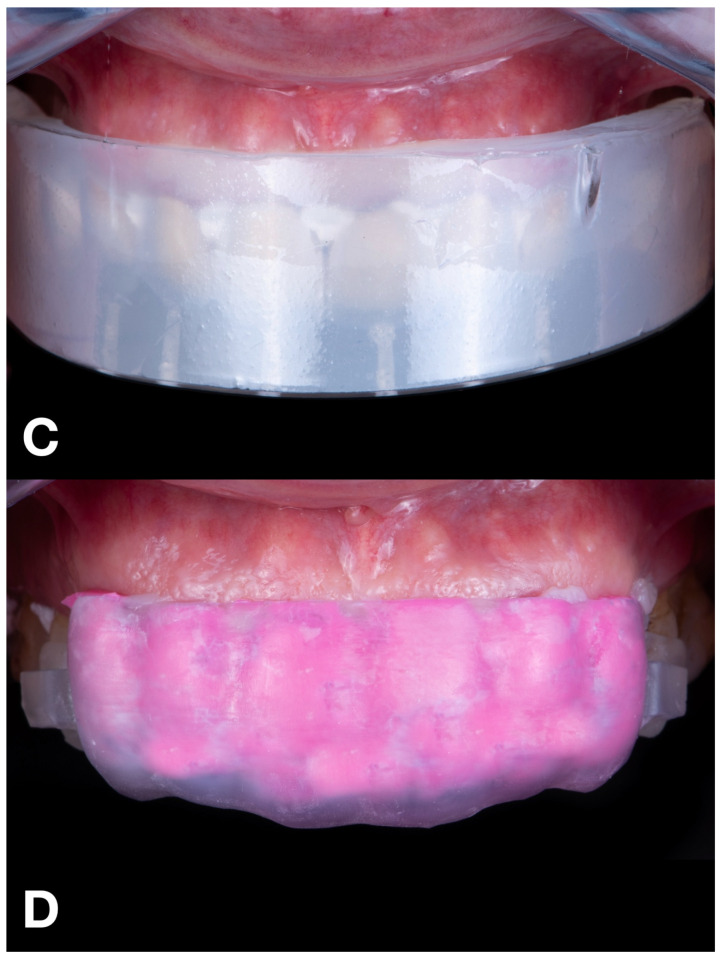
Mock-up matrices intraoral application for trial restoration performance, intraoral view: (**A**) condensation silicone matrix; (**B**) addition PVS silicone matrix; (**C**) transparent addition PVS silicone matrix; (**D**) CAD-CAM combination matrix.

**Figure 4:**
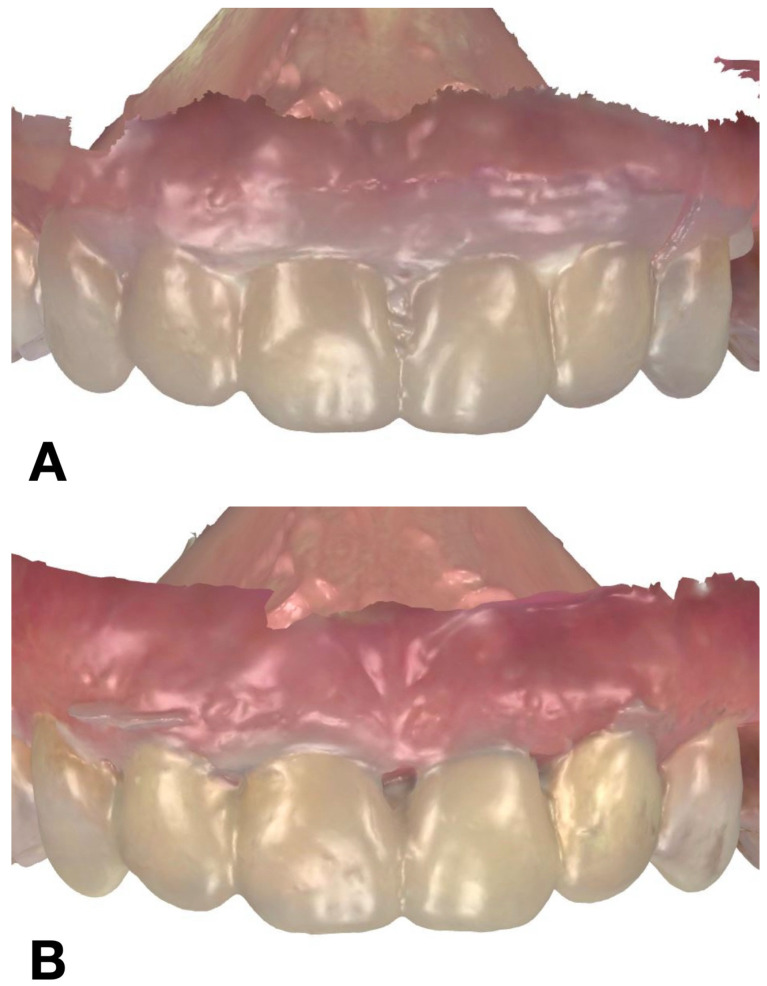

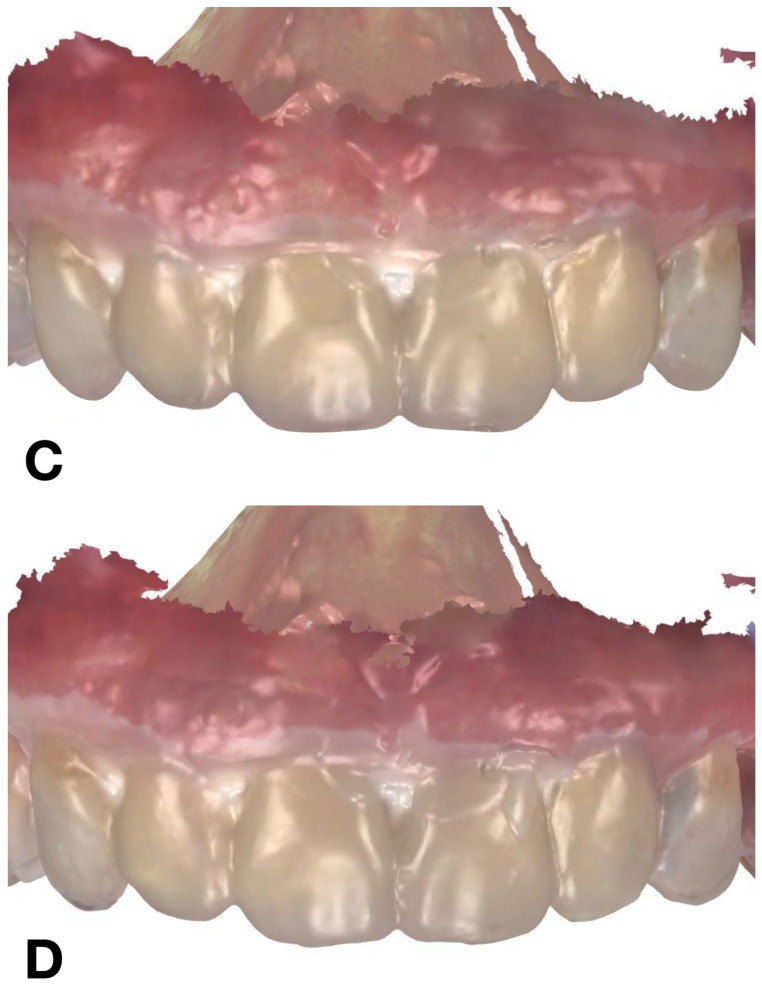
IOS of mock-up: (**A**) condensation silicone matrix; (**B**) addition PVS silicone matrix; (**C**) transparent addition PVS silicone matrix; (**D**) CAD-CAM combination matrix.

During a single clinical appointment, all four trial restorations were performed for each patient, following a standardized workflow (Figure 3). The intraoral scan STL file, previously recorded during the diagnostic appointment, was duplicated four times (once for each technique tested), and the area corresponding to the six anterior teeth was virtually trimmed. Each trial restoration was performed and scanned over the trimmed STL files (Figure 4).

**Figure 5:**
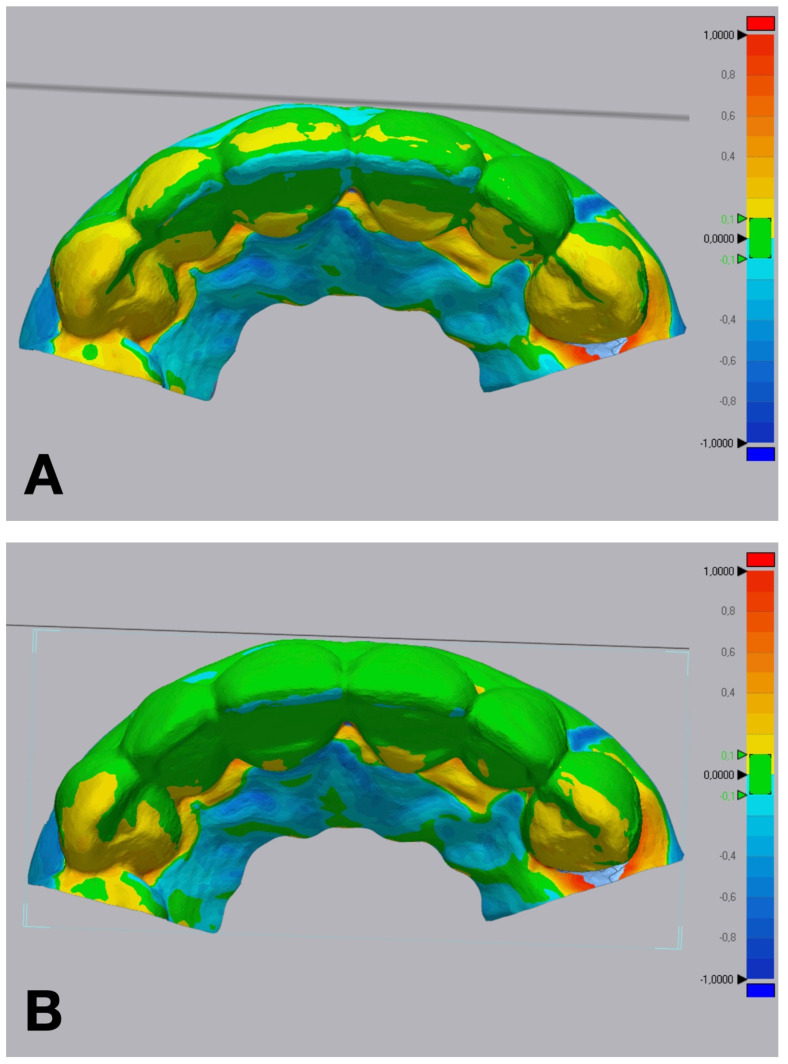

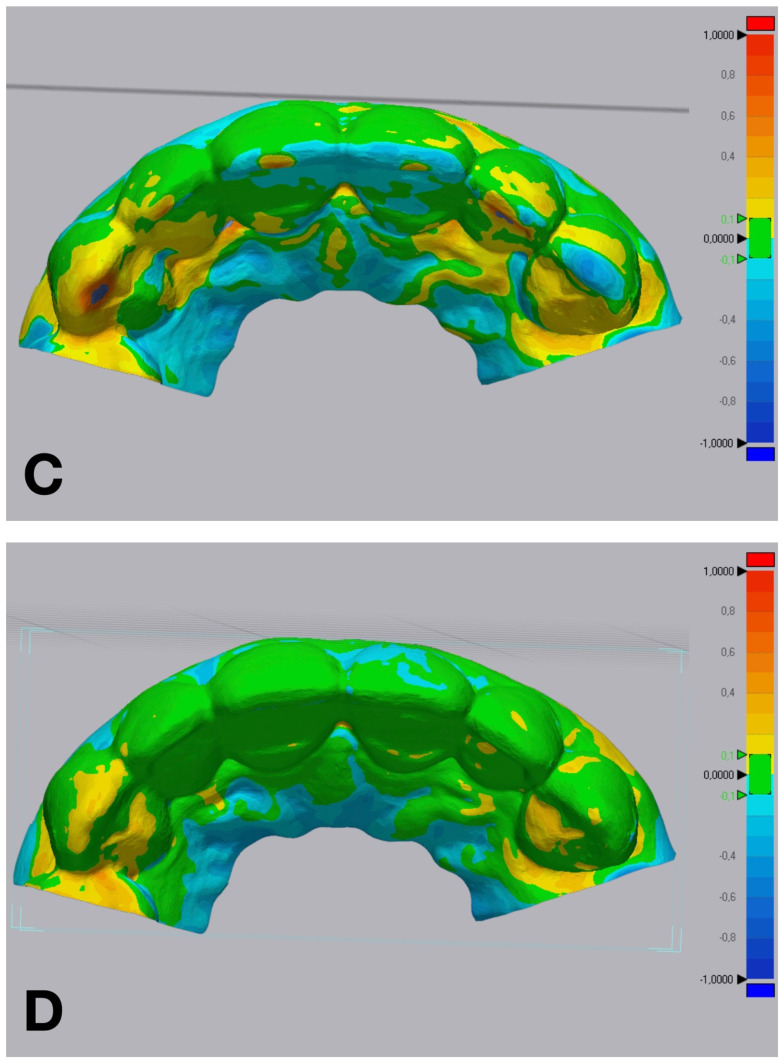
Volumetric discrepancies comparison within the diagnostic wax-up and the mock-up using a non-dental reverse engineering software (Geomagic Control X 2018.1.1, 3D Systems Inc., Rock Hill, SC, USA): (**A**) condensation silicone matrix; (**B**) addition PVS silicone matrix; (**C**) transparent addition PVS silicone matrix; (**D**) CAD-CAM combination matrix.

**Figure 6:**
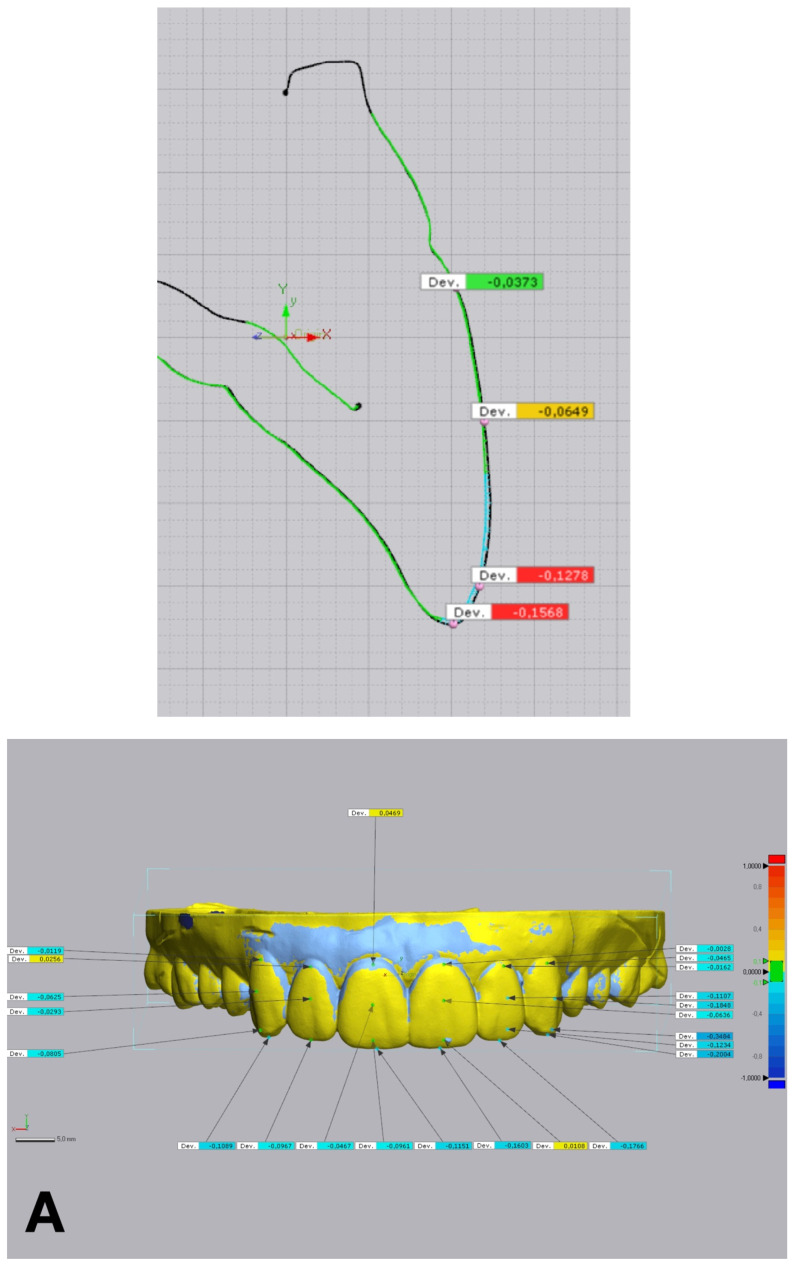

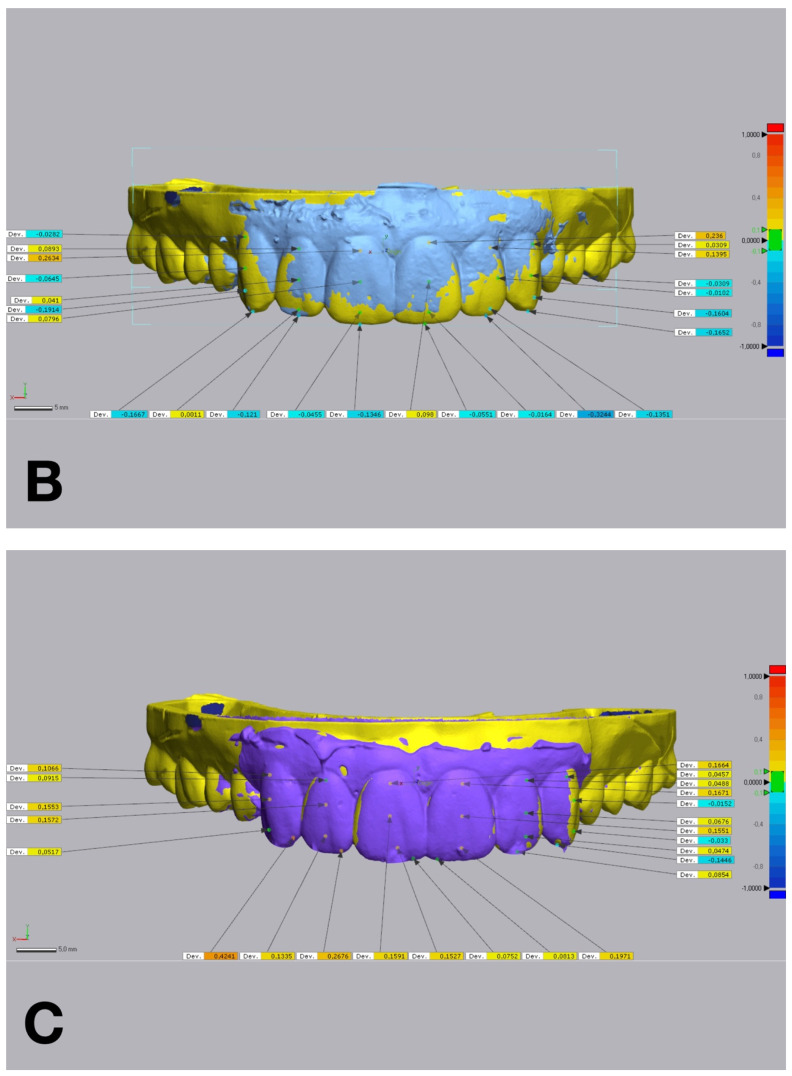

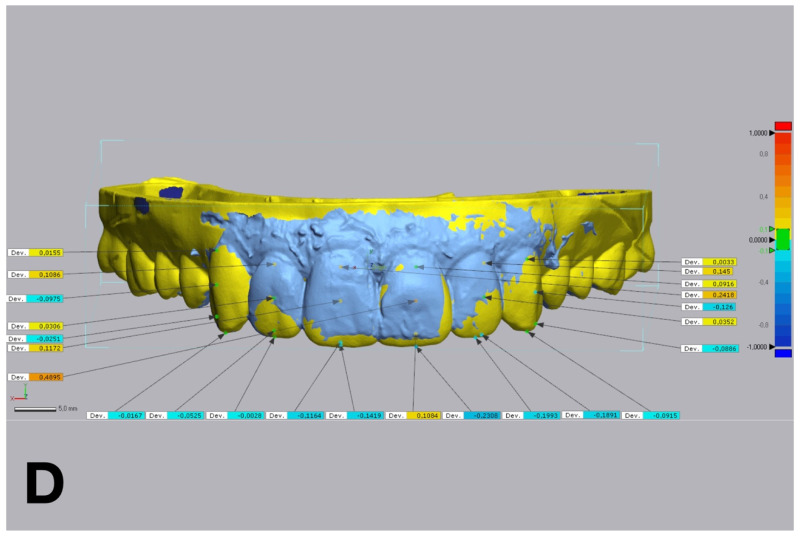
Linear discrepancies comparison within the diagnostic wax-up and the mock-up using a non-dental reverse engineering software (Geomagic Control X 2018.1.1, 3D Systems Inc., Rock Hill, SC, USA), calculated by measuring the distance at four points (cervical, medial, lower medial, and incisal) on the buccal aspect of each waxed tooth: (**A**) condensation silicone matrix; (**B**), addition PVS silicone matrix; (**C**) transparent addition PVS silicone matrix; (**D**) CAD-CAM combination matrix.

The accuracy of each technique was determined by evaluating the discrepancy between the IOS of each trial restoration scan and the reference STL file. Each trial restoration IOS was imported and aligned to the control reference STL cast of the waxed printed cast using the “best fit” algorithm of a non-dental reverse engineering software (Geomagic Control X 2018.1.1, 3D Systems Inc., Rock Hill, SC, USA). Dimensional accuracy was evaluated through volume and linear analyses. The root mean square (RMS) values of volumetric discrepancies between trial restoration STL files and reference STL files were calculated using a non-dental reverse engineering software (Geomagic Control X 2018.1.1, 3D Systems Inc., Rock Hill, SC, USA) (Figure 5). Linear discrepancies were measured at four buccal points (cervical, medial, lower medial, and incisal) on 6 teeth, from 13 to 23, with the same software (Figure 6). All the measurements were recorded in an Excel spreadsheet.

Due to the within-subject design and the nature of the interventions, blinding of the operator and participants was not feasible. The four matrix techniques varied in material composition and clinical handling and were therefore not visually or physically identical.

No changes to outcome measures were made after the trial commenced.

### 2.5. Statistical Analysis

The measured linear discrepancies for addition silicone (A), condensation silicon (CO), and hybrid technique (H), as well as the measured volumetric discrepancy for hybrid technique (H), did not follow a normal distribution (*p* < 0.05). Levene’s test also demonstrated a statistically significant difference between the measured linear and standard deviations of the volumetric discrepancies (*p* < 0.05). Consequently, non-parametric Kruskal–Wallis (K-W) and Mann–Whitney (M-W) tests were performed to assess statistical differences in the linear and volumetric discrepancies in the studied groups. The statistical analysis was conducted using a dedicated statistics software package (Statgraphics Centurion X; StatPoint Technologies Inc., Warrenton, VA, USA). Statistical significance was set at *p* < 0.05 with a confidence interval of 95%. All statistical analyses, including pairwise comparisons, were pre-specified prior to data collection.

## 3. Results

A total of 20 patients (12 females and 8 males; mean age: 34.7 ± 7.9 years) were included in the study. All participants met the inclusion criteria and successfully completed the trial restoration procedures. A total of 80 IOSs were analyzed from the four matrix groups, with 20 reference STL files serving as the control.

**Figure 7:**
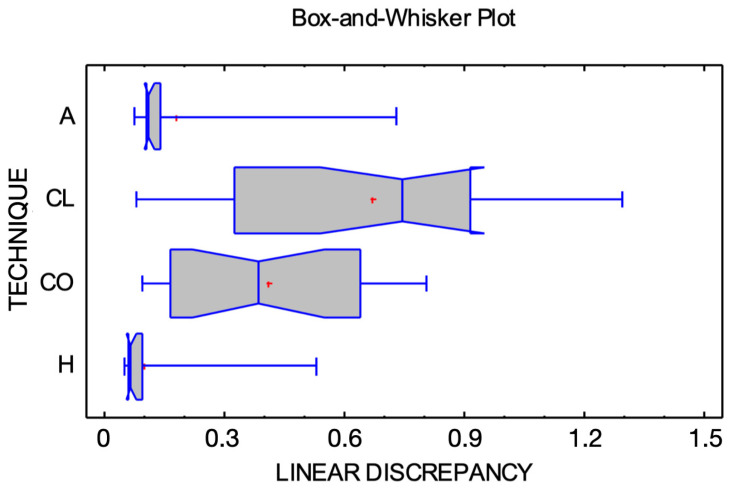
Box-and-whisker plot illustrating linear discrepancies measured in addition silicone (A), clear matrix (CL), condensation silicone (CO), and hybrid matrix (H) groups. All measurements are in millimeters. Median notches indicate differences among groups.

**Figure 8:**
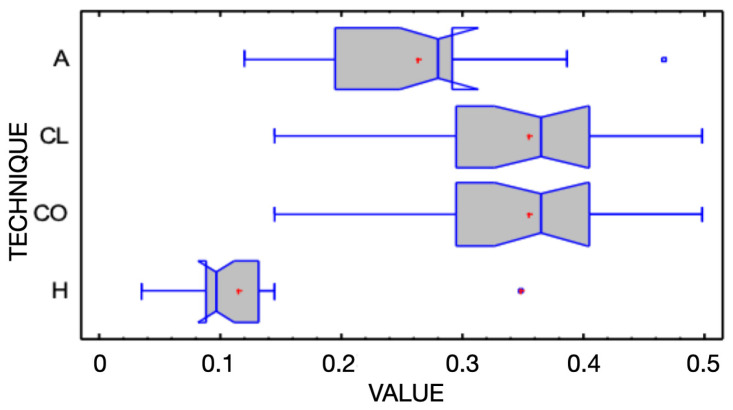
Box-and-whisker plot illustrating volumetric discrepancies measured in addition silicone (A), clear matrix (CL), condensation silicone (CO), and hybrid matrix (H) groups. All measurements are shown in millimeters. The box plot highlights the quartiles, median and potential outliers. Median notches indicate differences among groups.

Discrepancies between the linear and volumetric measurements were observed among all groups (pK-W < 0.05) (Table 1 and Table 2) (Figure 7 and Figure 8).

The CAD-CAM combination matrix exhibited the lowest volumetric discrepancies compared to the reference STL files, with a mean root mean square (RMS) deviation of 0.105 ± 0.012 mm. The addition PVS silicone matrix demonstrated comparable volumetric accuracy, with an RMS deviation of 0.123 ± 0.015 mm. The condensation silicone matrix exhibited moderate accuracy, with a mean RMS deviation of 0.185 ± 0.022 mm. The transparent PVS silicone matrix showed the highest volumetric discrepancies, with an RMS deviation of 0.256 ± 0.031 mm (*p* < 0.001).

Linear discrepancies were measured at four buccal landmarks: cervical, medial, lower medial, and incisal. The CAD-CAM combination matrix consistently demonstrated the smallest deviations across all points (cervical: 0.089 ± 0.011 mm; medial: 0.097 ± 0.013 mm; lower medial: 0.106 ± 0.014 mm; incisal: 0.112 ± 0.015 mm). The addition PVS silicone matrix performed similarly, with slightly higher discrepancies (cervical: 0.094 ± 0.013 mm; medial: 0.103 ± 0.014 mm; lower medial: 0.113 ± 0.016 mm; incisal: 0.119 ± 0.018 mm). The condensation silicone matrix displayed higher deviations (cervical: 0.152 ± 0.021 mm; medial: 0.161 ± 0.023 mm; lower medial: 0.175 ± 0.026 mm; incisal: 0.183 ± 0.028 mm), and the transparent PVS silicone matrix showed the largest deviations, particularly at the incisal edge (cervical: 0.202 ± 0.031 mm; medial: 0.218 ± 0.034 mm; lower medial: 0.234 ± 0.036 mm; incisal: 0.281 ± 0.041 mm) (*p* < 0.001).

Kruskal–Wallis and Mann–Whitney U tests revealed significant differences among the four groups in both volumetric and linear accuracy (*p* < 0.001). Post hoc pairwise comparisons indicated that the CAD-CAM combination matrix and the addition PVS silicone matrix were significantly more accurate than the condensation silicone matrix and the transparent PVS silicone matrix (*p* < 0.05). The transparent PVS silicone matrix consistently showed the lowest accuracy and the highest variability among all groups.

The CAD-CAM combination matrix achieved the highest accuracy due to its rigid structure and calibrated relining, minimizing deformation during the transfer process. The addition PVS silicone matrix also demonstrated high accuracy, attributed to its material stability and external resin shell. In contrast, the transparent PVS silicone matrix exhibited significant inaccuracies, particularly at the incisal edge, due to material shrinkage and variability introduced during sprue trimming.

No harms or unintended effects were observed during the study.

## 4. Discussion

This study assessed the dimensional accuracy of four techniques for fabricating and transferring diagnostic trial restorations, analyzing both volumetric and linear discrepancies. By evaluating distinct procedural workflows and material selections, the findings reflect real-world clinical practice and provide actionable insights for clinicians.

### 4.1. Clinical Significance

Among the tested techniques, the CAD-CAM combination matrix demonstrated superior accuracy in both volumetric and linear assessments. The rigid structure and calibrated space for low-shore PVS silicone material minimized deformation during relining, ensuring precise replication of the diagnostic wax-up. Features such as buccal openings and distal stops further facilitated visual control and fit assessment, making this technique particularly suitable for complex anterior aesthetic rehabilitations.

The addition PVS silicone matrix also performed exceptionally well, offering results comparable to the CAD-CAM matrix. The dimensional stability of PVS materials, combined with the reinforcement provided by the external resin shell, contributed to its reliability. However, the technique’s precision depended on careful handling during the putty-wash process, particularly during spacer placement and light silicone relining. These findings underscore the addition PVS silicone matrix as a practical alternative, especially when digital technologies are unavailable [17,18].

The condensation silicone matrix exhibited moderate performance, with higher discrepancies compared to the CAD-CAM and addition PVS silicone matrices. The inherent instability of condensation silicone, prone to shrinkage and deformation during curing, likely influenced these results. Additionally, manual trimming introduced operator-dependent variability, underscoring the limitations of this material for precision-demanding applications [19].

In contrast, the transparent PVS silicone matrix demonstrated the highest variability and lowest accuracy among the techniques. The reliance on sprues, which required trimming, introduced inconsistencies, particularly in critical areas such as the incisal edge. Material shrinkage further compounded these inaccuracies. While transparent matrices offer visual control during placement, their reduced rigidity and deformation susceptibility make them less suitable as preparation guides, although they remain useful for interim prostheses and aesthetic previsualization [20,21].

### 4.2. Methodological Considerations

This study employed a “best fit” algorithm within non-dental reverse engineering alignment software to quantitatively assess discrepancies, providing accurate volumetric and linear measurements. To minimize methodological bias, the same intraoral scanner was used for acquiring both diagnostic wax-up and trial restoration STL data [22]. Additionally, duplicating and trimming the same baseline STL file for each restoration ensured procedural consistency across all groups, further enhancing the reliability of the results.

Finally, the rationale for selecting different materials for each matrix type was based on widely accepted clinical protocols. Auto-polymerizing acrylic resin and light-cured flowable composite resin were chosen for their compatibility with specific matrices. This approach allowed for a comprehensive evaluation of the entire process, considering the combined influence of material properties, matrix design, and procedural handling on accuracy.

### 4.3. Clinical Applicability

Minimally invasive restorative procedures increasingly advocate for the use of trial restorations as guides for dental preparation, enabling the use of calibrated burs for accurate tooth reduction [3]. The findings of this study emphasize the critical importance of selecting appropriate matrix fabrication techniques to ensure accurate replication of the diagnostic wax-up and maintain correct prosthetic space for restorative materials.

The CAD-CAM combination matrix demonstrated the most reliable accuracy and clinical applicability for cases requiring both buccal and incisal coverage. Its rigid structure and digitally calibrated relining provide consistent outcomes, making it an optimal choice for precision-demanding anterior restorations. The addition PVS silicone matrix, while slightly less precise, remains a reliable option due to its dimensional stability and ease of use in conventional workflows [23,24,25,26].

In contrast, the transparent PVS silicone matrix should be used with caution when employed as a reference for dental preparation. Its reduced rigidity and the variability introduced by post-processing steps, such as sprue trimming, limit its precision in highly detailed cases. This technique is better suited for less demanding applications, such as interim prostheses or previsualization of final restorations.

### 4.4. Limitations and Future Research

This study has several limitations that should be considered. First, the fabrication and trial restorations were performed by a single operator, eliminating variability due to operator skill but limiting the generalizability of the findings. Given that the operator’s skill and experience are known to influence accuracy, future studies should evaluate the impact of operator variability to better represent diverse clinical scenarios.

Additionally, this study excluded emerging materials for matrix fabrication using CAD-CAM technologies. At the time of writing, low-rigidity 3D-printed resins for fabricating resin mock-up matrices did not meet minimal aesthetic standards and failed to transfer intricate details. As a result, these materials were omitted from the present analysis. Future research should explore the clinical potential of such materials as advancements improve their mechanical properties and ability to replicate fine details.

Finally, this study focused exclusively on maxillary anterior teeth and included a relatively small sample size. Future investigations should expand the scope to include posterior teeth and complex occlusal anatomies, offering broader insights into the clinical utility of these techniques across diverse restorative scenarios.

## 5. Conclusions

In conclusion, this study provides valuable insights into the accuracy of various diagnostic trial restoration matrix techniques. The findings underscore the importance of aligning material selection and fabrication methods with specific clinical demands to ensure precise outcomes in restorative procedures. As advancements in digital dentistry and material science continue to emerge, clinicians are encouraged to adopt evidence-based approaches that optimize both efficiency and accuracy, contributing to improved patient care and outcomes in prosthodontics.

## Figures and Tables

**Table 1:** Median (Q2–Q3) linear discrepancy of the studied groups. The *p* * column indicates Mann-Whitney (M-W < 0.05) comparisons between groups. All data are in millimeters.

	Linear Discrepancy	*p* * (<0.05)
1. Addition Silicone	0.11 (0.10–0.14)	X
2. Condensation Silicone	0.38 (0.17–0.64)	X
3. Clear Matrix	0.74 (0.32–0.91)	X
4. Hybrid Matrix	0.08 (0.06–0.10)	X

**Table 2:** Median (Q2–Q3) volumetric discrepancy of the studied groups. The *p* * column indicates Mann-Whitney (M-W < 0.05) comparisons among groups. All data are in millimeters.

	Volumetric Discrepancy	*p* * (<0.05)
1. Addition Silicone	0.28 (0.19–0.29)	X
2. Condensation Silicone	0.40 (0.39–0.48)	X
3. Clear Matrix	0.37 (0.29–0.40)	X
4. Hybrid Matrix	0.10 (0.09–0.13)	X

## Data Availability

The original contributions presented in this study are included in the article. Further inquiries can be directed to the corresponding author.
